# Periods and well-being: A focus group study to discuss how menstruation affects the well-being of women aged 18–40

**DOI:** 10.1177/17455057251362992

**Published:** 2025-08-19

**Authors:** Caroline Musulin, Natania Yeshitila, Joyce Harper

**Affiliations:** 1 EGA Institute for Women’s Health, University College London, UK

**Keywords:** periods, women’s health, fertility, infertility, menstrual cycle, menstruation

## Abstract

**Background:**

Menstruation is a central experience in women’s lives throughout their reproductive years, and in some cases, it can significantly impact quality of life. Investigating women’s attitudes towards their periods, how it may affect their well-being and identifying areas for improved education and support is crucial.

**Objectives:**

To explore women’s attitudes towards their periods, the impact on their well-being, their management strategies, and their experiences with menstrual education, along with suggestions for improvement.

**Design:**

Qualitative analysis of online focus group discussions.

**Methods:**

Participants were divided into two groups according to their age. Five focus groups were conducted for each group. Group 1 included 26 women aged 18–25 and group 2 included 29 women aged 26–40. Women were recruited online via social media to participate in a focus group to discuss their menstrual experiences. The discussions were transcribed and analysed using content analysis to identify key themes and commonalities.

**Results:**

Participants expressed mixed attitudes towards their periods, often highlighting the inconvenience to their daily lives, whereas some noted a sense of community among women. Overall menstruation negatively impacted various aspects of their well-being, from physical health to personal relationships. Participants employed a range of management strategies and overwhelmingly felt that their education inadequately prepared them for menstruation. Stigma emerged as a pervasive theme, influencing their attitudes, well-being, management approaches, and educational experiences.

**Conclusion:**

This study underscores the significant impact of menstruation on women’s well-being and highlights the urgent need for improved menstrual education and support. Recommendations include providing early, gender-inclusive education and ensuring there is support for those with difficult periods. The importance of support networks in managing menstrual health is also emphasised.

## Plain language summary

**Periods and wellbeing: A focus group study to discuss how periods affects women aged 18–40** Periods play a major role in women’s lives during their reproductive years and can significantly affect their quality of life. In total, 55 women took part in focus groups to discuss their menstrual experiences. This study explored women’s feelings about their periods, how periods affect their wellbeing, how they manage them, and their experiences with menstrual education. It also looked for ways to improve education and support. The research included two groups of women: one group aged 18–25 and another aged 26–40. Participants were recruited through social media, and focus discussions were analysed to find common themes. The results showed mixed feelings about periods. Many women found them inconvenient and said they negatively affected their physical health, personal relationships, and overall wellbeing. However, some appreciated the sense of connection having a period created among women. Most participants felt unprepared for their periods because their education on the topic was inadequate. They also shared how stigma around periods shaped their experiences and made it harder to talk openly or seek help. The study highlights the need for better menstrual education and support. It recommends providing early, inclusive education that addresses both boys and girls and taking a more comprehensive approach to periods. Building support networks and reducing stigma are also essential to help women manage their menstrual health more effectively.

## Introduction

Menstruation, or a period, is the cyclic shedding of the endometrial lining that occurs at the start of each menstrual cycle.^1,2^ Period lengths can vary but last on average around 5 days.^3^ Most women experience around 480 periods between their first period and menopause.^4,5^ Menstrual health is a vital indicator of a woman’s overall health and well-being.^1,6^ This article refers to “women” in the context of menstruating individuals; however, not all individuals who menstruate identify with their sex at birth.^7^

The presence of menstrual symptoms such as dysmenorrhoea, abnormal uterine bleeding (AUB), and premenstrual syndrome (PMS) can significantly impact overall quality of life (QoL).^8–10^ Dysmenorrhoea is defined as pain during the menstrual cycle^10^ and affects 70%–90% of people who menstruate and can affect their overall health and impact their ability to attend school or work and participate in social events, sports, and hobbies.^11^ Painful periods may arise from idiopathic causes or identifiable aetiologies like endometriosis and adenomyosis.^12^ AUB is characterised by changes to the volume, duration, regulation, or frequency of bleeds^13^ and is a condition that up to one-third of women will experience during their life.^14^

PMS has a global prevalence of ~47% and includes both affective and somatic symptoms^15^ during the luteal phase that usually resolve during menstruation.^16,17^ Symptoms may involve angry outbursts and depression, while somatic symptoms include abdominal bloating and weight gain.^16^ Although up to 90% of women experience at least one PMS symptom during their reproductive years, these normally do not significantly impact QoL.^18^

Premenstrual Dysphoric Disorder (PMDD) is a severe and often debilitating form of PMS affecting which causes intense emotional and psychological symptoms such as mood swings, irritability, depression, anxiety, and difficulty concentrating.^19^ These symptoms can interfere significantly with daily life, relationships, and work.

Menstruation has historically been, and continues to be, heavily stigmatised.^20,21^ This has manifested in various ways, including reluctance to discuss menstruation openly, the frequent use of terms like “sanitary towels” and “menstrual hygiene product,” which imply that menstruation is unhygienic, cultural myths labelling women as “impure” during their periods, and research showing that women often feel shame and self-consciousness about menstruation.^22–24^

The lack of education and normalisation of difficult periods restricts individuals from seeking help. Many women endure menstrual problems longer than they should due to the inability to speak openly about uncertainties, fears of being dismissed by healthcare professionals (HCPs),^25^ the view that its “just” a painful period,^26^ or feelings of shame.^22,27^ Stigma, secrecy, and the expectation to “cope” with painful periods contribute to the systemic dismissal of menstrual discomfort.^26^ Strict behavioural expectations to conceal menstruation and resource limitations contribute to negative experiences, where dissatisfaction with menstrual practices and management environments exist, along with feelings of disgust if participants feel they have failed to manage their menstruation discreetly.^28^

Menstrual education is essential for equipping girls and women with knowledge about their menstrual cycle and tools to manage their periods.^29,30^ However, in most countries, menstruation education is very limited or non-existent. A 2024 global report by the World Health Organization (WHO) revealed that only 39% of schools worldwide provide menstrual health education.^31^ This figure is even lower in primary schools, particularly in regions like sub-Saharan Africa. The report emphasises that the absence of menstrual education contributes to stigma, misinformation, and poor health outcomes among students.

In our studies with teenagers in the United Kingdom, they report at the most two lessons on menstruation – usually one at primary school and one at secondary school, but some did not have these.^32^ Recognising the critical importance of physical, relational, and menstrual health, the UK government made the Relationships, Sex, and Health Education (RSHE) guidelines a compulsory component of all school curriculums in 2019, ensuring parents could no longer withdraw their children from RSHE education.^33^ These guidelines, with specific regard to menstrual education, have since been revised, and a 2023 draft has been made public.^34^ The proposed guidelines include all original information, such as key facts about the menstrual cycle, implications for emotional and physical health, and how to manage menstruation.^34^ Additionally, they suggest including more comprehensive information, such as the distinction between normal and abnormal periods, conditions like Polycystic Ovary Syndrome (PCOS) and endometriosis, and other topics like fertility and menopause that evolve throughout the lifespan.^34^

The WHO report shows that millions worldwide are unaware or unprepared for menstruation before their first period.^31^ For instance, a study in Ethiopia found that less than half of the surveyed girls knew about menstruation prior to experiencing it. A 2018 report on young women aged 9–18, published by Plan International UK, found that one in four women felt unprepared for the start of their period, one in seven did not know what was happening, and almost half felt embarrassed by their period.^22^ A UK government call for evidence in 2021, including nearly 100,000 women aged 16 and over, revealed that fewer than one in five women felt they had enough information on menstrual well-being, and fewer than one in 10 had information on the menstrual cycle throughout the lifespan (e.g. menopause) and other gynaecological conditions.^27^

Previous studies by our team have addressed the age groups at either end of the reproductive lifespan (schoolgirls^32^ and perimenopausal women^35^) using focus groups to explore their attitudes and feelings towards their periods, the impact on their well-being, and how better support can be provided in formal education and beyond. In this study, we used the same protocol with women aged 18–40. The study was conducted in two groups according to age under the premise that women of different ages may have different views to menstruation. However, the results were almost identical and so in this article, the analysis for both ages have been merged and differences highlighted.

Focus groups were used as they are a valuable method for exploring women’s experiences as they create a space for open dialogue, shared reflection, and validation.^36^ This approach can also foster a sense of support and inclusivity, making it especially useful when researching sensitive or stigmatised topics.

## Methods

The reporting of this study conforms to the COREQ statement and is reported in the Supplemental Material.^37^

### The researchers

All three women involved in this project were cis women. This project was led by Professor Joyce Harper (BSc, PhD) who is a 62-year-old researcher who led all the focus groups and has extensive experience of running focus groups. The 18–25-year-old focus group study was conducted by Caroline Musulin who is a 23-year-old MSc student. The 26–40 year old focus group study was conducted by Dr Natania Yeshitila who is a 26 year old MSc student (MBBS), Obstetrics and Gynaecology. We decided on the age ranges as from our previous studies, we know some women start trying to get pregnant from their late 20s onwards^38^ and we wondered if there was a difference in experiences of menstruation between those who were not trying to get pregnant and those that were.

### Study design

This study was built on previous research^32,35^ on how periods affect the well-being of 15-year-old girls and perimenopausal women, respectively. All groups were asked the same four questions:

1. How do you feel about having a period?
2. How does your period affect your well-being?
3. How do you manage your period and menstrual cycle, and what support do you need (e.g. food, exercise, period trackers, work)? Do you talk to your friends or partner?
4. How could we improve education around menstruation, and how was your education at school?

These questions aimed to facilitate open conversations about both shared and individual experiences among group members, without influencing or biasing responses.

Ethical approval (project ID number: 9831/008) was obtained at University College London (UCL).

### Recruitment and data collection

Recruitment was achieved through a poster shared on the social media platforms of all authors, including Instagram, Twitter, Facebook, and LinkedIn. Information was also shared via word of mouth and personal contacts. The details included the intended participant demographic, the associated institution (UCL), the general study premise, contact information for inquiries, and confirmation of ethics approval. Recruitment was open from 10 May 2024 to 1 July 2024. Inclusion criteria were cis women currently experiencing an active menstrual cycle (e.g. periods) and aged 18–40 years. Women who did not speak English, gender diverse, and were outside the age groups were not included. The reasoning behind only including cis-gendered women was to explore menstruation as experienced within the context of female socialisation and biology. This allowed for a clear and consistent scope. Trans and non-binary people who menstruate have important experiences that deserve separate, focussed research. The women had no prior relationship with any of the researchers and no information was given about the researchers except that they were doing this project and their position in the department (Professor and MSc student).

Potential participants were sent the information leaflet and consent form by Caroline Musulin or Natania Yeshitila. The leaflet outlined the study’s premise, its voluntary nature, the option to opt out at any point, the use of recorded media, participant anonymity, procedures if a participant felt distressed during the focus group, and data handling. Participants needed to complete a consent form to receive the invite to the focus group, confirming they understood the information and still wished to join. Each participant was assigned a unique pseudonym to ensure anonymity and to protect their identity in the results and sent details of the zoom meeting. The zoom meeting was recorded to create a transcript and video and audio recordings were deleted.

Focus groups were conducted from 24 May to 5 July 2024 using the focus group guide (Supplemental Material) and lasted between 50 and 60 min each. By the fourth focus group for each age group, saturation was achieved, and further recruitment was deemed unnecessary. A total of 26 participants were involved in group 1 and 29 in group 2.

### Content analysis

Researchers discussed initial observations after each session and summaries were produced. Analysis followed Braun and Clarke’s inductive thematic approach,^39^ allowing themes to emerge organically from the data and ensuring authentic reflection of participants experiences which aligns with qualitative research principles, particularly those rooted in grounded theory or thematic analysis, where the aim is to remain open to participants’ perspectives rather than imposing pre-existing assumptions. By allowing themes to emerge from the data, rather than forcing them into pre-determined categories, the analysis stays closer to the participants’ actual language, meanings, and lived realities. This enhances the credibility and authenticity of the findings and helps ensure the research reflects the complexity and nuance of real-world experiences.

Transcripts were reviewed multiple times for familiarisation followed by coding using NVivo software (Lumivero, version 14). Initial codes, seen as the “building blocks” of sub-themes and theme,^40^ were organised by question to maintain contextual accuracy. Repeated sentiments across questions were preserved unless a response clearly pertained to another question. Codes were iteratively reviewed and grouped into sub-themes, such as “stigma” and “normalisation of pain,” which were further consolidated to broader themes like “the bad.”

Redundant or non-essential codes were excluded from the final analysis. We retained codes that reflected recurring patterns and excluded those that duplicated meaning already captured by broader or more representative codes, were either overly descriptive without analytical value, contextually irrelevant, or too minor to support theme development. Decisions were made through team discussion to ensure consistency and transparency. Although thematic analysis is largely descriptive, it is inherently interpretive, acknowledging the researchers influence on the final themes.^41^

The initial analysis was done separately for the two age groups. However, since there was large overlap between the results, the results were subsequently merged. To do this, several meetings were held between all three authors to discuss the data and decide on the final common themes and the differences. Quotes are numbered depending on the age group: 1 for ages 18–25 and 2 for ages 26–40.

## Results

The eight focus group discussions consisted of 55 participants. Because the focus group discussions (FGDs) took place online, participants could have been located anywhere in the world, however, given the main recruitment avenues were via the authors’ social media accounts, it is likely that most participants were located in the UK. Overall, the women felt passionately about sharing their experiences. A few women reached out after their session to thank us for the work we are doing and the personal value they gained from attending the session.

### Question 1: How do you feel about having a period?

Participants highlighted both positive and negative aspects of experiencing a period, and many oscillated between them. Negative elements mostly revolved around the inconvenience as well as the mental and physical toll of having a period, whereas positive elements often reflected sentiments of connection to other women and their own bodies. Two themes were found: *The bad: I wish it could end already* and *The good: We’re all in this together.*

#### The bad: I wish it could end already

Most women expressed at least one negative sentiment towards having a period. For many women, these were all-consuming, with some of their opening lines including: “*I absolutely hate it, and I’ve hated it since I was about 12 years old when it started*” [Cindy][2] and “*It’s like the bane of my existence every month*” [Rose][1]. A few women in the older group felt neutral about their period, and that it was “*just kind of. . . one of those things*” [Rachel][2].

Many women described their periods as a significant inconvenience and burden, both physically and mentally. The stress of managing schedules, ensuring the availability of menstrual products, and coping with symptoms led many to view periods as disruptive and inconveniencing in their daily lives.

I find it very inconvenient just to never know like, when it will come, how long it will be the pain, like what I’ll be able to do on it. [Aria][2]

I have to constantly be thinking, oh, when is it starting? When is it stopping? How am I gonna manage it? So I definitely think the kind of stress levels can come in there. [Eva][1]

This sentiment was particularly pronounced for those with irregular periods, as the unpredictability added to their anticipation and dread.

It’s just something that happens, basically, that dread of, you know it’s gonna come, I think, also, because sometimes it’s not always regular. . . And you’re like, then just kind of stuck trying to relax in your period, but also have lots of stuff to do. And that’s a surprise when it sometimes turns up, which is just awful. [Shirley][1]

Many participants highlighted both the physical and psychological toll of having a period, particularly in the days leading up to and during their menstrual bleed, and how these impeded on their daily activities. Among the older group, for some their period also served as a painful reminder that they were not pregnant.

I think for me it is also quite a nuisance, mostly. It has a huge effect, physically and mentally. [Susan][1]

Like every month it’s like, “Oh yeah, here it is again, just another reminder that I’m not pregnant.” [Cindy][2]

Several women turned to societal issues related to menstruation in shaping their attitudes, primarily expressing notions of stigma, shame, and feeling that they had to “hide” their period.

So initially when I first got my period about like 8 to 9 years ago, I think I was very ashamed of it which is probably something cultural. . . the women would always try to hide it. So I really grew up, hiding my period, hiding my pad under my books, hiding my cramps, and, and just like having to act normal. [Zoe][1]

I think there was a lot of shame in the early years, even though we had a lot of different resources in our school, and they provided free period products in every bathroom, there was still this big stigma. [Ella][1]

For some women, stigma was also viewed as a contributing factor to the normalisation of female pain, delays in seeking treatment, and poor provision of women’s healthcare. Several women explored these sentiments by reflecting on their experiences with HCPs where they felt dismissed and unheard.

Even when I go to the doctors and I tell them, and they’re like, oh, maybe you should like try reducing your weight, and I was like but I have done it though, so it’s very frustrating for me to deal with them. [Samantha][1]

The doctor told me as well like it will be like a 10-year process if you ever want to get like an endometriosis diagnosis. So, she basically told me, like, you just have to rough it out. [Sharon][1]

#### The good: We’re all in this together

Some women expressed ways in which having a period has impacted them in a positive way.

The most common positive sentiment was the role of menstruation as an invaluable biomarker. Women appreciated the reassurance it provided in several ways. Confirming they were not pregnant was deemed one of the most important among these.

Sometimes I feel a bit of a relief cause as a young woman you’re kind of told as, oh, if you get your period, then you’re not pregnant, and that’s a good thing, cause you shouldn’t be getting pregnant at such a young age, so I think, sometimes there’s that kind of relief from a pregnancy point of view. [Eva][1]

Sometimes a relief because I was fearing to be pregnant when I didn’t want to be pregnant, so that I welcomed my period in those occasions. [Esme][2]

And indicating proper bodily functions (e.g. ovulation) and providing reassurance of fertility.

I feel a sense of relief. I feel like everything is working, I’ve obviously ovulated, and things are in the right place. [Helen][2]

I think I’m happy when I get it, because it’s a sign of fertility, and I have a lot of friends that even at our young age they kind of not have their cycling like periodically. [Cara][1]

It could also provide clarity and relief by explaining prior adverse symptoms.

I always find I’m worse before my period, and but when I get my period, I’m kind of like, almost relieved in the way, because it’s like, that’s why I’m feeling like that or like it explains it. [Claire][1]

I find that it explains everything that’s been going wrong with my life the previous few days. Cause, for some reason I forget I have a period every month. So I’m like yeah, this is it. So then I’m like, it makes sense. [Lauren][1]

The second most common positive aspect was the sense of connection women felt, both with other women and their own bodies, reinforcing their sense of “womanhood.” For some, this connection offered a silver lining, turning shared challenges, like forgetting a menstrual product, into opportunities for bonding.

It also has a weird sort of, it makes me feel connected to other women or people who menstruate in a weird way. I don’t, I feel like they have quite a collective experience. That kind of makes you feel connected quite quickly, and like helps you bond a little bit I suppose. [Susan][1]

It gives me the sense of a womanhood. . . it makes me feel like a complete woman. [Hannah][1]

### Question 2: How does your period affect your well-being?

When asked about the impact of their periods on well-being, most women highlighted the negative effects on their QoL, such as disruptions to emotional and physical health, daily activities, and relationships. Unlike responses to question 1, few positive aspects were noted. Four themes emerged: *Mind and body – Why am I crying, Daily disruptions and unwelcomed shifts: Why am I eating all of this chocolate?, Interpersonal strain and guilt: I just turn into a bit of a monster, Unfulfilled hopes: Oh god! Another period!*

#### Mind and body: Why am I crying*?*

Most women reported both psychological and physical symptoms as significant impacts of their period on their well-being. For many, the psychological effects were pervasive, particularly during the premenstrual stage, influencing multiple aspects of daily life. Symptoms included reduced mood, reduced self-esteem, low energy, and reduce desire to be sociable.

The PMS is, I think, the worst thing of just like a week or 2, kind of like not feeling like myself, feeling really depressed, feeling really pessimistic. [Susan][1]

The week before, I am considerably emotional. So, I do believe I have PMDD, currently seeking diagnosis for that. But I am, so it ranges month to month, some months I’m incredibly suicidal. Others I’m just very upset, brain fog, nothing works, I hate myself. That type of mood. That’s the week before. But then, when I’m actually like on my period, I am I’m a non-functioning individual. [Charlotte][2]

These contrasted with the relief and positive feelings of recognising earlier symptoms as PMS.

I think, as Lauren just mentioned once I’ve got my period I will be like happier because I know that there is an explanation for that. I’m not going crazy, or I’m not getting depression. [Nicole][1]

There’s definitely like a feeling like when it, when the period is over, and you’re sort of going into your fertility bit. Like it, I feel lifted like I feel like, “Oh, my God, okay, I’m like, back. I’m normal again.” [Mia][2]

Physical symptoms, including pain, temperature fluctuations, heavy flow, acne, and bloating, were also commonly cited, with pain ranging from manageable to debilitating. Many women recognised the interconnection between well-being elements, like the combined physical and mental impact of acne during periods:

Acne starts coming up on my face. . . it makes me very stressed when that happens. [Cara][1]

Participants varied in their experiences of menstrual related pain, highlighting how variable women’s physical experiences can be.

The pain and how heavy I bleed just leave me to the point where all I do is sleep and cry, and I’m now at the point where I have to take codeine just to manage the pain when I’m on my periods. [Charlotte][2]

Because I use a hot water bottle, and because it’s so painful I have really bad burns on my stomach now . . . I can’t not have a hot water bottle for about at least 2 weeks, because it’s, I can’t deal with the pain. [Shanon][1]

Reduced self-esteem was often connected to the appearance of physical symptoms such as “bloating” and “feeling heavy.”

It impacts my confidence. Just right before the period even, maybe perhaps also with throughout just in terms of like this feeling of tiredness and heaviness that comes with it. So yeah, it does definitely impact my, my self-confidence, and also self-esteem. [Tara][1]

Bloating for me is one of the biggest things that impact my sort of wellbeing. I feel like, because suddenly like I feel like I look pregnant or like I’m like struggling to button up my jeans, and it’s so uncomfortable that that fate makes me feel like absolute crap. [Mia][2]

Low energy was often also associated with reduced abilities to concentrate and engage in daily tasks.

I think everything just feels a bit hard. You feel lower energy, less engaged, less able to concentrate, less interested. [Bailey][2]

I think the thing that affects me the most is the tiredness. I think all the physical symptoms aren’t great, but because it’s been like 15 years of having a period, that I just I’m used to it, so painkillers and stuff it’s manageable. But the tiredness really affects me. [Lily][2]

#### Daily disruptions and unwelcomed shifts: Why am I eating all of this chocolate?

Many women reported that their periods affected their eating habits, exercise routines, and work productivity. Some avoided leaving the house during their period, with some participants articulating that their periods can “ruin” their whole day.

I feel like a week before I get it I get these cravings, especially for sweets, and I’m like, Oh, my God, what is wrong like, why am I eating all of this chocolate? And then I get my period. [Olivia][1]

I also like, quite like running. And I feel like before my period my running is so bad, and I like just don’t have nearly as much energy. And it can be that can be quite frustrating. [Chloe][1]

Several women also spoke about their periods at work, with some feeling less productive, “disadvantaged,” and set back in their work because their period.

In some situations, can be a little bit embarrassing, especially at work, like I feel like I get really like frustrated easily, and quite often whenever I get frustrated, I’m a like a crier. So, then I almost feel like embarrassed if I’ve like, cried in front of like my work colleagues, and I’m just like, “Why have I just done that?” [Katherine][2]

There’s just like 2 weeks where I feel like I’m at a disadvantage most of the time. But then I try to make up for it. So, it’s sort of like this up and down monthly, which can get quite exhausting. So, I feel like with my period it’s more like I feel a bit disadvantaged. [Monica][1]

#### Interpersonal strain and guilt: I just turn into a bit of a monster

Several women expressed feelings of guilt and the interpersonal strain that their periods caused in their relationships with friends, family, and partners. Many also shared a sense of frustration and isolation due to the societal taboo surrounding menstruation, which made it difficult for them to feel heard or understood. Common sentiments included feeling particularly triggered or “ticked off,” experiencing heightened annoyance, and having more arguments than usual. Emotional burdens were further compounded by the lack of open dialogue, leaving them feeling dismissed and unsupported.

I think, the only time. . . I think I’m very agreeable with people, but that week before my period is the only time every month that I would have arguments with people, especially my partner. . . I just turn into a bit of a monster, even though I know why I’m doing it is, like, it makes me very ashamed of being in such a bad mood, or being so sensitive, which makes me, which causes a strain onto my relationship with my partner. . . I just I don’t know how to control it. [Sharon][1]

It’s like quite a taboo subject that people don’t talk about it. So, you kind of feel like you’re burdening people if you talk about it as well. Which I think can probably have a bit of a detrimental effect impact on your wellbeing too. [Lucy][2]

For a few these sentiments were also echoed in their experiences with menstrual healthcare.

I’ve seen six gynaecologists. Some of them have told me it will get better with age. I’m currently on the waiting list right now to go back to another gynaecologist, but I’m also considering going private, because I know something’s wrong with my body. [Charlotte][2]

#### Unfulfilled hopes: Oh god! Another period!

In the older cohort, for some feelings related to fertility and the emotional challenges of trying to conceive emerged and the sense of failure when their period arrived.

I think it’s when it’s affecting my wellbeing has been, you know, when you’re using it to, um like for fertility planning, you know, if if your cycle’s not regular or something’s out of the ordinary, then that has a real big impact, and you’re kind of waiting to see the next cycle what that’s gonna be like. So, um in terms of kind of fertility planning, that’s when I think psychologically, can have a really big impact. [Rachel][2]

But then, like I said, extra up, like mental health issues around like the TTC (trying to conceive). And then thinking about, “oh, God! Another period! Why hasn’t it worked?” [Olivia][2]

### Question 3: How do you manage your period and menstrual cycle and what support do you need (e.g. food, exercise, period trackers, work)? Do you talk to your friends or partner?

The women described several ways they manage their periods, including strategies and support systems. There was also an emphasis on the limitations and barriers encountered. Two themes were identified: *Practices and tools: I’m quite kind to myself* and *Support systems: A big part of managing your period.*

#### Practices and tools: I’m quite kind to myself

We specifically asked the women about food, exercise, period trackers, and work and they had varying responses. They also talked about products, such as transcutaneous electrical nerve stimulation (TENS) machines and pain killers.

##### Food

Some women talked about craving certain foods and gravitating towards them. Chocolate and carbohydrates were a common craving among many of the women.

In terms of food I definitely feel the need to treat myself more with chocolate and sweets. Things like that definitely help me get through. And I definitely have more cravings for them as well and I just kind of honour those cravings because I don’t think there’s any benefit to cutting them out. [Eva][1]

I eat a lot of chocolate, especially a couple of days before, I just get starving. And specifically, for like sweet treats. [Evelyn][2]

Some women managed their nutrition by avoiding certain foods around the time of their period as they felt this might reduce their cramps and other symptoms. This included coffee, dairy, spicy foods, and other unhealthy products and substitutes for healthier options.

I think the, definitely diet, just choosing to eat healthy like soups and veggies things like that it makes a really big difference. So just at least the week before my period, I do try really hard to eat like warmer, healthier things. [Sharon][1]

I get a lot of sugary food cravings, and I think I that happened all through my teenage years, and more recently, I’m trying to experiment with kind of staying disciplined and eating kind of round, balanced meals. [Bailey][2]

##### Exercise

Participants varied greatly in their approach to exercise throughout their menstrual cycle. Some said it did not affect them at all, so they carried on as usual.

Exercise, I don’t see a huge difference when I’m on my period. [Debra][1]

Some managed their exercise routine to accommodate how they felt before and during their period. Many women found light activities like yoga, Pilates, and walking beneficial for pain and mental health.

In terms of exercise, I would probably kind of change a bit of what I’m doing if I don’t feel like going to the gym. . . maybe try and go on a walk or something. [Eva][1]

Some exercised despite not feeling like it to help alleviate their menstrual cramps.

I really don’t feel like exercising on my period, but when I do and like, when I force myself to do it, it makes me feel better. So I’ve seen that over like the past couple of years. So I do try and do it, even though I really, really don’t want to. [Rose][1]

When I first start, I really can’t be bothered, but towards the end of my cycle, I feel like I’m ready to get a bit more movement, and it’s almost like I hibernate for a couple of days, and then I come out feeling like a new woman. [Beatrice][2]

And some preferred not to exercise right before and during their period due to concerns of “leaking” or discomfort.

I’m always conscious of going to like exercise classes or anything, because I’m I’m just scared that I might leak, so I tend to just sort of do sort of like low intensity, like going for walks, and just sort of yeah, being out the house. [Emily][2]

There was one time I was in the gym, and I just, I got a cramp, and I had to just sit on the floor, and it was the worst cramp, so I just sat on the floor crying, I was like, yeah. So, to save myself from the embarrassment, I I don’t, I don’t participate in anything that I usually do. [Charlotte][2]

##### Period tracking

Most women tracked their cycles to help manage them, mainly using digital apps, though some used other methods such as basal body temperature, or manual records like spreadsheets. Many women found tracking apps helpful for planning activities, aiding fertility journeys, and understanding emotional changes, which was often described as “validating.” Although most viewed the apps positively, opinions varied.

I really like being able to know where I am in my cycle when it’s gonna come. And I think, having that kind of expectation can make me manage my periods better. [Chloe][1]

I’ve actually found having a widget, so using the tracking app data as a widget next to the time on my phone, has been really great for me. So, when I look at the time, it also tells me I’m day 18 and I, and it also tells me what phase that is, and I love having that daily awareness of my period and what phase I’m in. [Rebecca][2]

Many women liked using the apps for management advice and insights into their symptoms.

It’s helped me kinda understand what’s happening in my body more and sort of know which effects different hormones and whatnot are having, at what time and kind of differentiate between what might be due to this and what might be due to different things happening in life. [Susan][1]

Through tracking, I managed to recognize why I’m feeling this way. [Ivy][2]

Several participants used the apps for contraception and ovulation tracking and a few women reported using them with their partners.

I have a more stable partner so I feel like it also helps. I mean both for me, I did it for myself, but also for the fact of like becoming a bit more active, and knowing how to, you know, manage that as well. So I’m using Flo and also natural cycles. [Tara][1]

I use Flo. I mainly use that because my partner can link up to it so he has it, and notifies him where I am in my cycle, and tells him like how I would be feeling and stuff which I feel like it’s really helped, and it’s helped him to know like to not pester me on certain days, or say something a bit more stupid. [Shirley][1]

Common frustrations with tracker apps included inaccuracies and subscription costs, leading some women to stop using them due to unreliable period predictions.

Sometimes 2 days early, sometimes 2 days late. That’s like a range of 5 days where I could have it. So it was always like, okay, I’m not gonna use it. [Olivia][1]

Some found them unhelpful or intrusive because the notifications, which alerted them of what symptoms to expect for the current stage of their menstrual cycle, became intrusive. Others were concerned about data privacy. This led to several women deleting the apps.

All the stuff happening around data from those apps [. . .] I don’t want my data being part of anything that’s going to become more oppressive to women. So, I did get rid of it [. . .] now I just write in my Google Calendar. [Olivia][2]

##### Work

Many women expressed a desire to offload work and stress right before and during their periods to better manage symptoms and allow time for rest. Although some, particularly university students, found their workload flexible enough to accommodate this, others, especially those in healthcare or with fixed commitments like exams, found it difficult to offload work. A few women were provided with annual leave during their periods called menstrual leave.

I guess in that sense I just try and maybe if I know that it’s happening, try and offload some of that stress onto another week, or maybe push things back . . . to allow myself more time. [Eva][1]

I work for the NHS so I don’t get days off here and there. Yeah, a lot of shift work a lot of like, especially sometimes I’ve been working the hospitals before that’s very fast paced, and that can be not very pleasant especially when you’re in like a lot of pain. [Shirley][1]

My office provide me the menstruation leave for one day. Especially for the first day of menstruation. [Jessica][2]

Despite work flexibility, many women still found it difficult to reschedule around their period due to its unpredictability.

I guess that just some, to some extent instil some stress as well, because it’s like, when is it gonna come? Is it gonna come on a busy week or not? So busy week, am I gonna have time to like take few hours to just lie down or not? [Eva][1]

Some women felt comfortable requesting time off for their period, especially when their superiors were women whom they found more approachable.

She was also a woman, my manager, so that also helped. But I would just text her if I needed like the morning of or like the day of, because my cramps were that bad that I just could not get out of bed. So I just let her know like, hey, I know this is TMI, but, like my cramps are too bad right now. I can’t like, I can’t be present at work. [Monica][1]

My boss actually suffers quite significantly with her period. So, she, I’ve struck gold really, she totally gets it, and there’s no questions asked. [Emma][2]

Women with predominantly male colleagues felt frustrated by the inability to discuss their periods openly for fear of making their colleagues feel uncomfortable and assuming they did not understand.

I work in quite a male dominated environment and they, any talk about periods and men like instantly shrivel up and don’t want to hear anything about it [. . .] it’s seen as unprofessional, it makes people feel uncomfortable, which I also don’t agree with, cause I said, like all women have it, like, don’t, you know, we should be able to talk about it in a more open setting. I agree it’s work, so maybe in a more professional sense, but like it does affect our day to day, it does affect my mood, it does affect my ability to do work, so I wish I could talk about it a bit more openly. [Isabella][2]

Some women preferred to “keep quiet” and “push through,” feeling uncomfortable advocating for time off due to their period.

In terms of work, I would say, I find that quite hard to manage, or I still haven’t really figured out how to support myself with that because there’s definitely a bit of a taboo subject around it. I don’t think I’d feel comfortable mentioning it to a manager, if I’m struggling that day, I would just prefer to keep quiet, which isn’t great. . . It shouldn’t be like that these days. [Catharina][1]

A few women talked about the practical challenges of managing their periods at work, especially the availability of period products and disposal options. Some felt supported by their workplaces, like those who had access to free period products. But some said there was room for improvement.

I worked last summer, I worked like in an office with all men, and it was just me, and I had my period one day, and there was no period bin in the toilet, and I was like, oh, my god, what am I gonna do? And then I just, I felt quite uncomfortable. . . I feel like that’s still definitely something that needs to be worked on, like I feel like men just like don’t speak about it at all. [Clair][1]

##### Products

Many women mentioned using products like heating pads, hot water bottles, painkillers, menstrual products, and birth control to manage their cycles, primarily for pain relief.

We have these heating pads. . . you stick them on your stomach and they really help me. It’s like the one thing, especially in winter. And yeah, I, I always use them. [Catharina][1]

Other women found the use of TENS devices extremely helpful.

I got Myoovi, which is like the TENS technology, and I found I found it great [. . .] I’ve actually found it works amazingly. I it’s got to the point where I don’t actually need to take paracetamol and ibuprofen, and I just use the Myoovi. And it’s quite discreet so I can put it, like I can use it at work, I can put it like under my work trousers and it just seems to do the job. It seems to work as a distraction, and and my cramps are not bad at all, and then I can use it overnight as well. So that’s actually been a huge blessing for me, I’m glad I discovered it. [Isabella][2]

Additionally, other methods of managing pain which were mentioned included regulating bowel habits to prevent constipation, and sexual intercourse.

If I’m anywhere near constipated my pain is just so much worse. So, having a really good control of my bowel movements is really important. [Lauren][2]

Some women found supplements helped with managing periods.

I’m very into like holistic care, like Inositol, like I take Ashwagandha, certain things which I noticed have helped me regulate my periods. [Aria][2]

#### Support systems: A big part of managing your period

Support networks were central to each woman’s experience in managing their period. Many women mentioned that talking to others, including friends, family, partners, and community members, was an integral component of managing their periods. Although some sought practical support, like sharing period products, the focus was mainly on receiving emotional support and having open conversations. Women emphasised that speaking to others helped them to share the “burden” metaphorically. They appreciated having someone to talk to, whether to rant, have open conversations about shared experiences, connect with others who were also on their period, or to warn others about upcoming shifts in mood and encourage them to be extra patient. Friends were the most frequently mentioned source of support.

I’m quite open about it with my parents like I will just announce, like no one annoy me because I am due my period and my dad and my mom are just like, okay, fine, everyone’s quite calm about it. I talk to my friends about it. We’ll have a bit of a rant about it. [Shirley][1]

Talking to my friends and other women about it really helps, and everyone’s very understanding usually cause everyone knows how it feels. [Lily][2]

Syncing cycles was a common topic among the women, often forming the basis for many conversations.

[My girlfriends and I] just keep discussing it and keep talking cause our cycles are kind of in sync where each of us has like a week, I guess. So we’re always talking about it like one of my friends goes, oh, my god, I’m having the worst day ever! And one of my other friend goes, is it your time now? And she’s like, yeah, it is. So we’re always talking about it. [Samantha][1]

Partners were also recognised as key sources of support and understanding in managing periods.

Even with my partner, it’s been a year now. . . he’s very patient with me, and I really appreciate at least the understanding that oh, I’m on my period so this might be the week that I’m extra sensitive. So please be a little bit more patient with me, and we’ve worked through it now. [Sharon][1]

I’m in a relationship with a woman so obviously she has a period too. So, in that kind of way as well like, then you can kind of like, relate more, and have like a bit more of like an understanding so like of when even if like, we’re like on together, or like separately [. . .] And it just is a bit more easier, because, you know, like, even like when we’re, I’m going around hers, or she’s coming around mine. We mostly know that we’ll probably have something like pads or tampons that we need like, so that’s kind of like nice to kind of like, be able to relate in that way. [Katherine][2]

Although most participants expressed positive sentiments about finding support from others, several highlighted that opening up about their periods varied greatly depending on who they were speaking to, and support from close individuals and the community was not always guaranteed.

I talk with my friends, not with my partner. . . I don’t know, I am, maybe it’s because he’s a man so like it’s like, I don’t feel comfortable, or I would say that it’s like I don’t feel he’s understand, he will understand me like in whole aspects. [Stephanie][1]

Some women felt misunderstood or judged due to insufficient knowledge about periods from others, including family, colleagues, and partners. This lack of understanding hindered empathy and support.

He does sometimes get some facts quite wrong, like when I told him [. . .] I said, my uterus really hurts, and he asked me if I was pregnant, and I was like, “Well, you have a uterus all the time, not just when you’re pregnant.” So, I do, I do think men are still very clueless about what’s going on. [Helen][2]

Some women felt unsupported by their families, particularly when they were one of the few still experiencing a period.

If I spend like holidays with my family, and then everyone is post menopause there are no trash cans in the garbage in the toilet. And then I’m like, Oh, what am I gonna do with this now. [Lauren][1]

I was just like; I can’t keep doing this and people just wondering why I’ve locked myself up. I’ve become bit more open with people. So even just my family, my little brother, I’ve had to like just make him more aware that periods, this is what happens and like, I’m probably the worst case you’ll get, but it’s real and this is how to deal. [Charlotte][2]

Others highlighted that stigma acted as a barrier to managing their periods, as it prevented open communication and hindered the development of a strong support system.

I wanted to say in terms of like support and kind of talking to people, which is also, I think my motivation to participate in this, because I think there’s so little communication about periods, and it is still so stigmatised. And I think it’s so important to talk about it. . . It affects half the population like we said, there’s so many things like it affects you mentally, it affects you physically. And why shouldn’t you talk about it? [Susan][1]

### Question 4: How could we improve education around menstruation?

From this discussion, the women told us about their education and made many suggestions on how it can be improved. All women said that their education about periods was inadequate and there were many gaps in their knowledge, so they had to educate themselves. They also discussed how outside school, improvements need to be made and how they have educated themselves. Four themes emerged: *A serious lack of education: Mine was non-existent, We need to teach what is abnormal, We’ve had to educate ourselves*, and *Educating health professionals.*

#### A serious lack of education: Mine was non-existent

At best the women had received two lessons, one lesson in primary school and one in high school. If they did receive education, usually only a normal menstrual cycle and period was taught, and many key topics were omitted. There was a unanimous call for boys to be taught in school.

Mine was non-existent. I didn’t have anything at all in primary school. In secondary school, we learned about how babies are made in the womb. That is not menstrual health education. It was very much like, “This is the science of like how an egg gets fertilised” [. . .] I genuinely like, there’s the Mean Girls meme about like, “Don’t have sex, because you’ll get pregnant and die.” That was my reproductive health education. [Amelia][2]

They agreed that education should begin in primary school, before puberty, to reduce fear and ensure all girls are well-informed.

I remember being in primary school on a school trip, and one of the girls had her period. . . she was quite young to have her period, and we were going swimming, and she couldn’t swim because she was too young to use a tampon. . . and I remember there was a lot of gossip. . . I look back now, and I’m older, and I just think how awful for her. That must have been a really difficult experience. So definitely, yeah, more education, younger. [Sophia][1]

They also wanted repeated, age-appropriate instruction throughout their school years to better support development at different stages.

Because of my experience, I’m gonna make like, make an effort when I have kids to go through things with them like even at an earlier age, like, start the education from a younger age. Not when you’re just about to start your period like one year away from start your period, or some some people might have, some girls might have already started their period by the time we had that that lesson. Because I hear now from people that I know that, you know, the age is getting younger and younger. [Andrea][2]

It’s still very beneficial to be educated about it, even if you’ve already started your period because there’s so much more you can always learn. [Eva][1]

They desired an education that prepared them for self-management of their menstrual cycles, whether through emotional support or lifestyle advice.

I think education at school could be a lot more holistic, managing all the different symptoms and the importance of kind of maintaining your exercise routine and being conscious of the food choices that you make, it’s just super important. So, you feel kind of in control of the experience. [Bailey][2]

The priority should be to teach the practical application of these hormonal changes: how might we expect these changes to affect our mood, social battery, physical performance, and academic performance throughout the month. [Megan][1]

They believed it was important to convey that menstruation is a natural process that does not always require medical intervention. They disagreed with the prescription of the contraceptive pill as a solution for every woman.

I think we should explain what is physiology and what is also pathology related to menstruation and normalise everything, and also maybe stop hyper medicalisation of periods. Because if you think women are having periods for most of their lives, they can’t be taking drugs all these years. And it’s not okay, you know. I mean, of course, if you have a condition, and if you’re really struggling, yeah, you have to [. . .] even the pill as the gold standard to fix all issues. That’s not for everyone, and it shouldn’t just be given. [Esme][2]

The women emphasised the need for menstrual education to cover fundamental topics like female anatomy, menstrual products, the physiology of the menstrual cycle, and factors affecting conception. They emphasised that comprehensive menstrual education is crucial for normalising periods as a natural process, thereby removing stigma and shame and allowing periods to be discussed freely.

The first point of change in education could come from just acknowledging the fact that periods are like a normal thing that happens to literally almost every single person, every single female identifying person in their, in your lives. [Samantha][1]

It all starts with education, like the formative years of how you come to understand about periods, and I think being separated in school classes by gender is really detrimental because it kind of reinforces the fact that this is something that is not to be spoken about in front of a certain group, and also what that does for people that are non-binary and trans. You know, it’s kind of isolating them from different conversations. But yeah, I think importantly, everybody should be educated about it together. [Rebecca][2]

I grew up in a different country, in Italy. . . the education there, sex ed, any kind of education along these topics is so bad, it’s actually like, so sad how bad it is. It’s so taboo to talk about periods still to this day to be honest. [Tara][1]

All women agreed that classes should not be segregated by gender, believing mixed sessions are crucial for boys’ education. Although some saw value in single-sex classes to allow students to speak more freely, they still felt mixed sessions were essential to improve overall understanding. They suggested this could help boys understand and support their friends and future partners through menopause.

I definitely feel like the boys should not be separated, like it’s something they’re gonna have to deal with. . . They’re gonna end up with a partner or know someone that has a period, whether it be a younger sister, a daughter eventually. And it’s like they’re not gonna know what to do. They don’t understand. [Shirley][1]

The boys and girls got split up, which I thought wasn’t. . . I don’t know, I feel like it’s really important that the boys were there as well because it would mean that it reduces the taboo, the stigma. We could have also talked about it within our class instead of it being separated. I thought that was kind of bizarre and actually not very helpful. [Chloe][1]

There was also discussion about who should deliver education. They wished for accessible, accurate information sources and stressed the need for properly trained educators to deliver these lessons effectively.

Sometimes when I’m watching something like TikTok I’m like, is this just kind of like, is it legit? So, having something like that where it’s more easily accessible. But it also has the backing that you know it’s legit would also be helpful. [Lucy][2]

At one, one point health class was taught by, like the male gym teacher who should not have ever, you know, tried to teach this at all. [Olivia][2]

#### We need to teach what is abnormal

Participants felt that their education was usually limited to basic facts, covering how many days a period could last for and having a cycle lasting roughly 28 days. They did not recognise or seek help for abnormal periods.

When I started, my periods was painful, really painful. And but I tried to tell people around me like my friends, teacher, or even my mom. She was like, I just need to deal better with it and I’m just overreacting, being too sensitive and all that. So yeah, it took a long, long time to get across that it’s painful. [Camilla][2]

I think a big thing, I would say, for improving it would be talking about what is normal and what to do if your period is not normal, right? So, to talk about that, but also that like, if your period seems normal, that also doesn’t mean that you are 100% healthy. It was one of the things that bugged me about infertility. I thought, I have a you know, pretty regular, very heavy period, surely that means I’m super fertile. [Olivia][2]

A few women later discovered that what they were taught was incorrect.

I’m gonna be completely honest, I’m 25 now. So up until 2 or 3 years ago did I find out I had a 3rd hole? I had no idea. So that just shows how great my all-girls school education was. [Hazel][1]

I was told as well that you can get pregnant on any day, even during periods as well. So, I think there is a lot of misinformation out there. [Rachel][2]

They expressed frustration that it did not adequately cover crucial aspect of women’s physiology, including conditions like endometriosis, PCOS, and PMDD, leaving women unprepared to distinguish between “normal” and “abnormal” symptoms. The participants felt very strongly that this was missing from the education they had received.

In terms of what I’d like to see education wise, is definitely just more awareness of the differences in women’s periods [. . .] what we experience when we have periods, because it’s not, no ones are the same at all. And if you have, you know I can’t remember, PMDD, that’s catastrophic, and we need to be educated around that. If you are in debilitating pain, that’s catastrophic. People need to know these are the things that people are dealing with monthly, sometimes biweekly, sometimes erratically, because they don’t know when it’s gonna happen. [Lauren][2]

I don’t think personally that there is enough education so that the young girls know what is normal. You know it’s not normal to be so pain, in so much pain that you can’t get out of bed. That’s not, that’s not being a woman, that’s not having a period. That’s, there’s something probably not right. [Cindy][2]

Many believed normal physiological processes, like vaginal discharge, were abnormal. The lack of education on how external factors, such as stress and contraceptive methods, can impact menstrual cycles was another point of confusion.

I didn’t know that like there were stains throughout the month, and, like acidic with like the acid would like dye my underwear different colours, like I thought there was something really wrong with me and stuff. [Kate][2]

I think, education on kind of period adjacent or period related things like, what’s the effect of being on the pill? How is that going to affect your period, for example. [Sophia][1]

And they were rarely taught how to use period products.

I remember probably my second period freaking out, having like a tampon for the first time, “Am I allowed to pee with it in? How do I pee?” You know, not understanding what that was, and like holding it in until someone like my older sister came home, and I could ask her. [Olivia][2]

I remember, like using tampons wrong for a while, and them being quite painful, and that’s quite dangerous. [Catharina][1]

One woman shared a distressing experience where she was mocked by both her peers and educator after submitting an anonymous question about how to use a tampon.

We had this like box where you put anonymous questions you had, and I remember, I put a question in about not knowing how tampons worked and like where they went, and I was totally confused. And I remember my question got read out loud, and the teacher was like, how does someone not know how to use this? There’s three holes, there’s one in the middle, like it’s just that one. It’s that easy. And I remember being so embarrassed. The whole classroom was laughing, like, who put that question? And I was like, right, okay, this is terrible. [Chloe][1]

They also described how shame and stigma surrounding periods made them feel unable to discuss or manage their periods openly, even in simple tasks like changing period products.

That feeling of like being in like a mixed school, and having to like run, find your bag, put a tampon up your school jumper, and then like run back to the loo, and that being like this sense of secrecy involved in it, which I feel like if it had been more overtly spoken about like that sense of shame, that was like being taught into us. [Mia][2]

You should also learn to address this myth and stigma that comes with menstruation. They should learn to address it. They should, while teaching the children they should address this myth and stigma that comes with menstruation. [Hannah][1]

#### We’ve had to educate ourselves

The participants felt frustrated by the lack of adequate school education, prompting them to educate themselves about their menstrual cycles. Many turned to books, apps, websites, and social media, but often encountered misinformation.

With the introduction of, you know, social media and the Internet, I mean, it’s a wonderful source of information. But I think sometimes it’s too big, and there’s a lot of misinformation. [Melissa][2]

There’s like social media accounts that I follow now and they give you kind of tips for like things like what might be helpful for the pain and like being more aware of what you’re eating and things like that. I think that’s really useful. [Claire][1]

A few women recounted witnessing peers who had not received support outside of school and as a result had a negative experience tainted by fear and shame.

One of my friends. . . their parents didn’t talk to her, and she was like, I’m so terrified, I just don’t want to bleed out. [Samantha][1]

Some women also took on the role of educating others, driven by their own struggles to obtain gynaecological diagnoses.

I also went to a girl’s school for high school so there was a lot more conversation between friends and educating each other about things, which I think definitely helped. [Sophia][2]

In terms of my period, it was definitely, like Debra said, more my mom and my grandmother kind of taught me. So I remember when I first had it, I kind of knew what it was and knew what to do. [Susan][1]

#### Educating health professional

They believed better education is necessary for HCPs, so women and girls have adequate support when seeking care.

I feel like if we can educate GPs in this country. Somebody please! Then we can get their understanding to a level where young females can go to them, and they feel confident that they’re going to be listened to and taken seriously. [Emma][2]

Women felt unsupported by the NHS, struggling to get their concerns heard and facing delays in gynaecological diagnoses like endometriosis. They were frustrated by minimal, impersonal treatment, and difficulty finding alternative sources of information.

When I went with severe period pain, it was just go on the pill, go on the pill, go on the pill. [Emma][2]

I started getting really bad periods when I had, like my 1st period, it was awful, and I’m going to the doctors, and they were literally like you need to be on contraception now. That’s it. . . I got really badly depressed, I was crying all the time. Whenever I went back they were like. . . you need to go on something else, you can’t be having periods like that. . . I was 15, and now I realize it’s probably my hormones just trying to work itself out. And it’s just yeah, like there was no education from that side of it, and neither was there from school. There was none of it. [Shirley][1]

The inadequate education had broader societal impacts, particularly on men. Women linked the lack of understanding in men to boys’ poor education in schools.

All the GPs being male doctors that you speak to, dominates, and that that sense of perpetual dismissal by a male doctor being like, “Yeah, yeah, it’s normal.” And you’re like, “Do you even do you even know?,” you know. So, I think that sense of like, if they had been taught as children in mixed classes, with periods, understanding what’s normal and what isn’t, then they would then have a better understanding of a range going forward. [Mia][2]

## Discussion

This study explored the attitudes of women aged 18–40 towards their periods, the impact on their well-being, management strategies, and experiences with menstrual education. Our findings, alongside those of partner studies,^32,35^ enhance understanding of menstrual health across the lifecourse. Participants consistently reported that menstruation was a central concern, influencing various aspects of their daily lives. Inconvenience, unpredictable emotions, stigma, and a lack of understanding from others were frequently reported.

Management strategies varied, with many women using support from family, friends, partners, and workplaces. However, discrepancies between women’s educational experiences and their desired knowledge were evident, suggesting formal education inadequately prepared them for their menstrual journeys and contributes to stigma, misinformation, and a reliance on external sources for basic menstrual knowledge. Addressing these gaps is essential for improving healthcare support and public policies related to menstruation.

### Attitudes to periods

Our results agree with others that many women view their periods as difficult and painful,^8–19,42^ suggesting many have yet to find effective strategies to mitigate period-related discomfort.^43^ Several participants in the older age group experienced periods as a distressing reminder of unsuccessful conception attempts, highlighting the emotional impact, and need for adequate support.^44,45^

Stigma further compounded participants’ negative perceptions, aligning with literature on how menstrual stigma silences women’s health issues, normalises pain, and leads to inadequate healthcare and diagnostic delays.^25,26,46^ Many women reported a sense of being dismissed by HCPs, consistent with the Department of Health and Social Care’s 2021 call for evidence,^47^ and further contributing to negative attitudes. This highlights the urgent need for improved healthcare communication and support.

Despite these predominantly negative experiences, some women shared positive or neutral views of their periods. However, the relief described by a few participants, due to perceiving their period as a sign of ovulation and fertility, is scientifically incorrect, pointing to a concerning gap in menstruation education and indicating insufficient understanding of menstrual cycles.^48,49^

### Impact on well-being

The emotional and physical burden of premenstrual symptoms was a prominent theme, with participants likening their experiences to a rollercoaster of emotions.^50,51^ Our findings confirm established research on the impacts of PMS and dysmenorrhoea on QoL,^10–12^ as well as related behavioural changes like fluctuations in appetite.^52–56^

Many women did not recognise their symptoms as premenstrual until their period arrived, delaying coping strategies, and worsening the impact. This reflects a broader societal tendency to dismiss sometimes severe and concerning symptoms of PMS and painful periods, as merely a normal by-product of “just a period.”^26^ As a result, these findings may point to a problematic internalisation and normalisation of pain and other concerning symptoms among young women.^22,57^

The most common physical impact on well-being was menstrual pain, with a significant proportion of women finding the pain unmanageable and, in some cases, debilitating. Current research shows that many women experience moderate-to-severe dysmenorrhea.^10–12,58^ Alarmingly, a 2019 systematic review of 24 studies of individuals with dysmenorrhea found that only 11% of young women reported seeking medical advice for their pain.^59^ This study reflects those findings and underscores the need for healthcare providers to identify and address these issues in affected individuals as they may pose a significant threat to young women’s QoL.^8^

Positive relationships are widely regarded as a pillar of well-being,^60^ yet several participants noted that period-related mood swings and irritability negatively impacted their relationships. This was also found in our study with 15-year-old girls, where many said they would not socialise around the time of their period, often because of feelings of anger.^32^ This highlights the need for further research into how period-related symptoms influence relationships and women’s overall well-being.

Stigma and shame associated with periods led some to isolate themselves or present “a front” in social interactions. Physical symptoms, like premenstrual bloating and acne, were linked to body dissatisfaction and mental health issues, reflecting societal pressures on women to conform to ideals of beauty and thinness.^61,62^ The guilt associated with increased appetite and cravings during the premenstrual period also contributed to feelings of shame and perceived failure in controlling their bodies.^63^

Participants’ dissatisfaction with healthcare was another key finding, echoing concerns raised in previous studies.^27,64^ Many felt dismissed by HCPs, often tirelessly advocating for themselves to receive diagnoses for conditions such as endometriosis and PCOS. The low number of women seeking care for severe PMS or PMDD, despite the significant impact on well-being, suggests dissatisfaction with previous healthcare encounters or perceived stigma.^65^ Educating healthcare providers and improving support, particularly for those experiencing severe mental health difficulties such as PMDD, is crucial.^66^

### Managing symptoms

Participants used various strategies to manage their periods, including tracking apps, self-care, dietary changes, adjustments to daily routines, use of pain relief, and social support.

Most women who experienced dysmenorrhoea relied on over the counter pain relief medications, yet research suggests that these are often insufficient.^67^ A 2021 study found that many women undertreat their pain or use ineffective methods, indicating a gap in knowledge about effective pain relief strategies.^67^ This aligns with participants’ reports, highlighting the need for improved education on pain management.

The use of tracking apps were frequently reported by the participants. They were valued for educational insights, validating emotions, aiding the planning of daily life, and supporting fertility journeys. However, several women also expressed frustration with the inaccuracy of the apps’ predictions of their period and their imposed paywalls which we have reported previously.^68^ Given the growing popularity of these apps, addressing these issues is crucial to ensure that women have access to accurate and affordable tools. The existing literature which shows that tracking apps develop women’s health literacy, combat stigma, and improve self-management.^69^ However, a subset of participants discontinued using these apps, with a few citing concerns over privacy, or preferring other simpler methods of tracking. Given the global low menstrual health literacy levels,^70^ the potential of these apps to educate and empower women is significant, as they offer an accessible way to learn about the menstrual cycle and reproductive health.^71^

Although many felt their workplaces were supportive, this was expressed by those who had predominantly female colleagues or female managers. Many women face challenges due to the nature of their jobs, discomfort in requesting time off, or a tendency to push through the pain, a sentiment shared by many participants.^72^ More work is required to ensure that all women have proper support, regardless of who they work with, by raising awareness of periods and menstrual health.^27^ The negative impacts of PMS on occupational productivity such as missed days off work are highlighted in previous studies, reinforcing the importance of policies including the British Standards Institution (BSI) guidance which advocates for menstrual leave, access to menstrual products, flexible working hours, and hybrid working which can be used during the peak of symptoms.^64,73–75^ Recent legal changes, such as the amendment to Section 80F of the Employment Rights Act 1996, which allows employees to request adjustments to working hours and conditions, could also offer improvements to women’s experiences in the workplace. Ensuring these practices are widely implemented can significantly improve women’s workplace experiences and their overall well-being.

Social support, particularly from female friends, was a significant factor in managing symptoms, aligning with previous literature.^76^ Although many women reported support from their partners, they also expressed frustration in their lack of understanding. This linked to a similar lack of understanding from male colleagues and managers, highlighting a need for greater awareness, and education across genders, starting in schools.^32^

### How can we improve education?

It is not surprising that almost every woman expressed dissatisfaction with their menstrual education, since lessons on periods ranged from 0 to 2 in their whole school life. Although we appreciate that some women might not be able to remember exactly how many lessons they had at school, this finding agrees with our recent focus groups with 15-year-old girls from England.^32^ Our findings highlight the need for early, continuous menstrual education, starting before menarche and reinforced throughout primary school. A 2024 study revealed that one in five respondents lacked formal education before menarche, a concern given trends of earlier menarche in girls.^76^ The current RSE guidance suggests that menstrual education should begin before the end of primary school by age 11, but this may be too late given the trend towards earlier menarche,^4,33,34^ perpetuating the cycle of inadequate menstrual education.

Although the new RSE curriculum has introduced period products and education in schools, there is still significant progress to be made, particularly regarding the breadth of menstrual health topics covered.^33,34,77^

It is important that we educate women about normal and abnormal anatomy, and physiology of the menstrual cycle at schools. Many women recalled distress due to a lack of understanding of their own anatomy and the physiological changes associated with their periods.^22^ A study of over 4000 participants found that half of those with significant dysmenorrhoea would not seek medical help, believing it to be a “normal” symptom of menstruation.^66^ When their periods changed following life events, such as stress or the discontinuation of contraceptive methods, they were left confused as they had not been educated on what to expect. It is also essential that menstruation education presents menstruation without bias so that young girls can experience their periods positively. Negative associations with periods can have profound and long-term impact on women’s perceptions of their bodies and menstrual health.^78^

Since some women struggled with understanding what was normal, this prevented them from seeking medical care and contributes to delayed diagnoses and treatment for conditions such as endometriosis, which can take up to 10 years to diagnose.^50^ Their frustration at not being taken seriously until after receiving a diagnosis, highlights the dismissive and paternalistic approach often encountered in healthcare and the need to address menstrual health awareness within the medical community more effectively. Participants emphasised the need for educating HCPs to take menstrual health seriously,^30^ as participants felt the NHS fell short in providing support, adequate information, and opportunities for discussion.^79^

Participants frequently mentioned that their educators lacked proper training, a concern echoed by 80% of UK teachers who felt additional training would have been beneficial.^80^ Gender segregated menstrual education also emerged as a significant concern which was also highlighted in our study with school girls.^32^ Previous research indicates that little has changed in the last two decades in improving the menstrual education boys receive.^13,19,32,81^ Many participants reflected on how the lack of education for boys perpetuated a cycle of misunderstanding, stigma, and shame that affected them later in life, a finding echoed in the literature.^82^ This gap in education affects not only the experiences of women but also the understanding of male teachers, managers, and HCPs, contributing to negative interactions and a lack of support. This gap persists as boys become men, affecting them into their midlife when their partner is going through menopause.^83^

Additionally, there is a notable gap in the understanding of fertility and reproductive health, with many women having misconceptions about conception.^84^ Our studies show that in the United Kingdom^85–88^ topics such as endometriosis and PCOS are not taught in schools. This can impact their well-being and family planning. Women in this study stressed that formal menstrual education should be comprehensive and inclusive, covering not only the basics but also the variety of experiences a woman may have. Participants called for accurate sources of information, open conversation, and normalisation of periods within society. This is exactly why the International Reproductive Health Education Collaboration has produced a number of resources to support teachers teach these topics.^88,89^

### Limitations

The recruitment via social media and word of mouth may have introduced sampling bias and in our experience, usually attracts a similar demographic to the researchers. The participants may have previously had notably negative menstrual experiences. This could impact the generalisability of our findings as they may not represent the wider population. This study only included cisgender women and further studies need to be conducted on gender diverse individuals.

Some participants may have felt uncomfortable expressing views that differed from the rest of the group due to conformity bias. The reliance on self-reporting introduces the risk of recall bias or exaggeration, which could result in inaccuracies in the data and the conclusions drawn from it. Finally, researcher bias must be acknowledged, as data analysis is inherently interpretive. Although efforts were made to minimise bias by systematically building codes before expanding to sub-themes and themes, there is still the possibility that the authors’ personal perspectives and prior knowledge influenced the process, potentially skewing the emphasis placed on certain pieces of evidence over others.

## Conclusion

This study completes a four-part project highlighting the profound impact of periods on women across their reproductive lifetime, from school age to perimenopause.^32,35^ Our findings demonstrate that menstrual education is insufficient, leaving women unprepared, leading to misinformation about menstruation symptoms which can lead to delayed care and diagnoses. Holistic, early, and gender inclusive education is essential for building menstrual health literacy and challenging the stigma around menstruation.

The study also highlights the persistent stigma and misunderstanding of periods which women face in all areas of their lives, including the workplace, relationships with partners and peers, and with HCPs. Reforming menstrual education and healthcare practices is essential to better support women’s menstrual health, including revising the RSE curriculum to ensure comprehensive coverage and ensuring supportive workplace policies are fully implemented.

Finally, employers should adopt the BSI workplace guidance to create supportive work environments for women.^74^ Implementing these can help tackle the menstrual stigma that exists in the workplace and ensure that women are appropriately supported.

## Supplemental Material

sj-pdf-1-whe-10.1177_17455057251362992 – Supplemental material for Periods and well-being: A focus group study to discuss how menstruation affects the well-being of women aged 18–40 Supplemental material, sj-pdf-1-whe-10.1177_17455057251362992 for Periods and well-being: A focus group study to discuss how menstruation affects the well-being of women aged 18–40 by Caroline Musulin, Natania Yeshitila and Joyce Harper in Women's Health
